# Does Patient History Influence Capsular Contracture? An Exploratory Analysis with Machine Learning

**DOI:** 10.1007/s00266-026-05919-8

**Published:** 2026-05-29

**Authors:** Thomas M. Johnstone, Daniel Najafali, Jennifer K. Shah, Justin M. Camacho, Chancellor Johnstone, Rahim S. Nazerali, Gordon K. Lee

**Affiliations:** 1 Division of Plastic and Reconstructive Surgery, Stanford University School of Medicine, Stanford, CA USA; 2 Carle Illinois College of Medicine, University of Illinois Urbana-Champaign, Urbana, IL USA; 3 Drexel University College of Medicine, Philadelphia, PA USA; 4 Air Force Institute of Technology, Wright-Patterson AFB, Dayton, OH USA; 5 Department of Plastic Surgery, University of California, Irvine, Orange, CA USA

**Keywords:** Alloplastic reconstruction, Artificial intelligence, Breast, Breast implants, Breast reconstruction, Capsular contracture, Complications, Contracture formation, Implant, Implant-based reconstruction, Implant capsular contracture, Machine learning, Mammaplasty, Outcomes prediction, Postoperative complications, Supervised machine learning

## Abstract

**Background:**

Capsular contracture (CC) is a frequent and distressing complication of breast augmentation and reconstruction. Although numerous patient-, surgical-, and implant-related risk factors have been proposed, reliable population-level predictors remain inconsistent across studies. This study evaluates whether administrative medical history, as encoded by ICD and CPT codes, contains sufficient predictive signal to identify patients at risk for CC using machine learning.

**Methods:**

Patients were queried from the Merative^TM^ MarketScan® Research Databases from 2003 to 2017 with CPT codes for implant-based breast reconstruction and augmentation. ICD codes were then used to identify all events and conditions of a patient’s history. Hyperparameter-tuned random forest models were combined with multivariable logistic regression models to validate risk factors for the development of CC.

**Results:**

A total of 112,489 patients were included, and the rate of capsular contracture was 9.55%. In total, 4,825 common conditions and procedures were included as features in the model. The random forest’s error rate was 9.54%. Prior CC was the most important variable in the model. When removed, model accuracy decreased by 0.0011% (*p* <0.001). Removing prior irradiation as a feature decreased model accuracy by 0.00014% (*p* < 0.001). In line with the previous literature, prior CC (OR = 2.47, *p* <0.001) and irradiation (OR = 1.16, *p* <0.001) were significantly associated with CC development in a multivariable logistic regression model.

**Conclusions:**

Administrative medical history alone demonstrated limited predictive utility for CC development. Although established associations such as prior CC and irradiation were confirmed, their incremental contribution to predictive performance was negligible. These associations do not meaningfully enhance a clinician’s ability to reliably predict which patients are at increased risk for CC.

**Level of Evidence III:**

This journal requires that authors assign a level of evidence to each article. For a full description of these Evidence-Based Medicine ratings, please refer to the Table of Contents or the online Instructions to Authors www.springer.com/00266.

## Introduction

Breast augmentation is one of the most sought-after surgical procedures in the USA for both reconstructive and aesthetic purposes. Given that over 1.8 million breast augmentations were performed in 2019 following at least a 20% increase in procedure frequency since 2015, significant attention has been dedicated to monitoring complication rates post-procedure [1]. One frequent complication that impacts aesthetic outcomes and quality of life after implant-based breast procedures is capsular contracture. The prevalence of capsular contracture has been reported to be between 2.8 and 20.4%, depending on factors such as radiation therapy, implant surface, and placement [2–4]. Capsular contracture is the result of an overactive inflammatory response that causes fibrotic tissue to surround the breast implant, which can yield significant pain and deformity [5].

Despite capsular contracture being a common complication, its pathogenesis remains incompletely characterized and likely is a multifactorial complication of surgery with several possible contributing factors, including inflammation, biofilms, genetic predisposition, operative techniques, and radiation [3, 6–9]. While the literature remains heterogeneous regarding the exact incidence of capsular contracture, it remains clear that this is a complication that has significant ramifications for patients and requires further investigation [10]. Capsular contracture is a leading cause of dissatisfaction and a frequent cause of reoperation in breast augmentation patients [11]. Therefore, it is imperative that surgeons are equipped with an understanding of the underlying pathophysiological basis and clinical implications of capsular contracture to deliver optimal results and quality care to their patients.

Although numerous patient-, surgical-, and implant-related factors have been proposed, the relative contribution of these variables remains inconsistent across studies, and reliable population-level predictors have yet to be established. Previous work has suggested that elevated odds of capsular contracture may be observed in patients with a history of capsular contracture, irradiation, or silicone (as opposed to saline) implants. Patient age, lower implant volume, utilization of a surgical bra, longer duration to follow-up, postoperative hematoma or seroma formation, subclinical infection, and bacterial contamination have also been raised as potential contributors to capsular contracture [12–14]. Even so, the literature remains mixed on key modifiable risk factors for capsular contracture, including prepectoral versus subpectoral implant placement [12, 13, 15, 16]. Notably, the identification of plausible contributors in individual patients does not necessarily translate into reliable population-level prediction, particularly when using administrative claims features.

Despite extensive research efforts to clarify causality and mitigate risk, capsular contracture continues to be the most common long-term complication of implant-based breast reconstruction and augmentation. More evidence is needed from emerging scholarship, which should leverage innovative methodologies to identify reliable risk factors. Few studies have used machine learning to assess capsular contracture development, with notable limitations in the number of cases evaluated, single-surgeon derived cohort (e.g., limited generalizability), homogeneous populations, and few input features [17, 18]. Amidst this landscape, our study utilizes a machine learning algorithm (i.e., series of hyperparameter-tuned random forests) to identify any outstanding medical history characteristics in patients undergoing implant-based reconstruction or augmentation that may predispose them to capsular contracture.

## Methods

### Patient Selection and Cohort Construction

Patients’ de-identified data between 2003 and 2017 were queried from the Merative^TM^ MarketScan® Research Databases [19]. The database contains inpatient and outpatient claims information from 182,915,708 unique patients. Using Common Procedural Terminology (CPT) codes, patients with implant-based breast reconstruction and augmentation were identified. International Classification of Disease (ICD) and CPT codes were utilized to identify all events and conditions of a patient’s medical and procedural history, as well as whether any patient had capsular contracture.

### Machine Learning Model and Statistical Analysis

A supervised machine learning approach using random forest models was fitted to predict capsular contracture development. Several applications have been assessed within plastic and reconstructive surgery and in other fields of medicine using this machine learning technique [20–23]. The random forest machine learning algorithm leverages ensembles of classification trees. A series of hyperparameter-tuned random forests was fit. The out-of-bag classification accuracy, permutation importance, and significance metrics were computed for the final model. To create a semi-parsimonious model, only diagnoses and procedures occurring in fewer than 1,000 patients were excluded. Finally, a multivariable logistic regression model with features of previous capsular contracture and prior irradiation was developed to validate established risk factors for the development of capsular contracture.

A threshold of *p* <0.05 was used as the threshold for statistical significance. All statistical analyses were performed using R (R Foundation for Statistical Computing, Vienna, Austria) (version 4.1.0) software in the Rstudio (version 4.3.2) environment. [24]

## Results

### Cohort Characteristics

Table 1 summarizes the cohort characteristics of patients in the model, with 10,752 patients having capsular contracture and 101,747 not experiencing capsular contracture. The mean age for patients without capsular contracture was higher (capsular contracture: 49.17 years, SD ± 8.44 vs. did not develop capsular contracture: 49.40, SD ± 9.08, *p* <0.001). The rate of capsular contracture in the cohort between 2009 and 2014 ranged between 23 and 27.8%.

**Table 1:** Characteristics of the study cohort (*n* = 112,489) in the Merative^TM^ MarketScan® Research Databases who underwent implant-based breast reconstruction with or without subsequent capsular contracture. Entries are frequency (percentage) unless otherwise specified. Statistical analyses conducted include chi-squared, Shapiro–Wilk, and Wilcoxon–Mann–Whitney tests

Characteristic	Experienced capsular contracture (*n* = 10,752) No. (%)	Did not experience capsular contracture (*n* = 101,747) No. (%)	*P* value
Age Mean (± SD)	49.17 (± 8.44)	49.40 (± 9.08)	<0.001
Year of reconstruction			
2007–2008	1464 (13.6)	17958 (17.6)	<0.001
2009–2010	2792 (26.0)	22852 (22.5)	
2011–2012	2984 (27.8)	25026 (24.6)	
2013–2014	2516 (23.4)	22081 (21.7)	
2015–2016	986 (9.2)	138240 (13.6)	
Region			
Northeast	2200 (20.5)	18307 (18.0)	<0.001
North Central	2063 (19.2)	21979 (21.6)	
South	4204 (39.1)	40702 (40.0)	
West	2058 (19.2)	18400 (18.1)	
Unknown or unspecified	217 (2.0)	2359 (2.3)	

### Random Forest and Multivariable Regression Modeling

There were 112,489 patients included, and the rate of capsular contracture was 9.55%. A total of 4,825 common conditions and procedures were included as features in the model. Table 2 summarizes the features of the classification accuracy and misclassification rate of our model in comparison to an ideal model. The random forest’s error rate was 9.54%. Incorporation of extensive historical diagnostic and procedural features improved predictive performance by only 0.01% over baseline incidence. A prior diagnosis of capsular contracture was the strongest predictor in the model. When removed, model accuracy decreased by 0.0011% (*p* < 0.001). Removing prior irradiation as a feature decreased model accuracy by 0.00014% (*p* < 0.001). Prior capsular contracture (OR = 2.47, *p* < 0.001) and irradiation (OR = 1.16, *p* < 0.001) were significantly associated with increased risk of capsular contracture development in a multivariable logistic regression model.

**Table 2:** Depiction of an ideal model versus the model developed in this study

Model Comparison	Classification Accuracy	Misclassification Rate
Model developed in this study	90.46%	9.54%
Ideal model	99%	1%

The percent of patients with capsular contracture in this cohort was 9.55%. The best performing random forest model improved on this rate by 0.01%. Therefore, it improves on simply guessing that all patients would not develop capsular contracture by 0.01%. A model with valuable predictors would improve on guessing by a larger margin. This suggests that the past medical history is not a valuable predictor of capsular contracture.

## Discussion

Capsular contracture is a frequent and challenging complication of implant-based breast procedures. As a result, plastic and reconstructive surgery literature has consistently explored the etiology of and risk factors for capsular contracture. The most commonly identified predictors are previous capsular contracture, surgical site infection, prior irradiation, implant characteristics, implant plane, and incision type [16, 25]. While many of the studies performed to identify these potential risk factors have been retrospective in nature and heterogenous, very few studies have tried to apply machine learning principles to the complexity of capsular contracture prognostication despite these algorithms’ proven utility in outcome prediction for breast reconstruction [26]. Amidst this landscape, this study leverages such a model to investigate which aspects of a patient’s history place them at increased risk for capsular contracture. Additionally, this study validates previous patient-specific factors believed to influence capsular contracture development using a large national cohort. The study’s findings highlight that, across over 4000 patient-specific input features, approximately 500 were observed to have high variable importance. Despite their significance in the model, the prediction accuracy of the generated machine learning model was similar to the incidence of capsular contracture in the cohort. Consistent with prior findings, the variables with highest importance of prior history of capsular contracture and irradiation were observed to be significant predictors of capsular contracture in our logistic regression model.

Capsular contracture likely reflects the interaction of immunologic, mechanical, microbial, and patient-specific factors not recorded in insurance claims data [27–30]. A more accurate predictive model would likely require integration of biologic markers, operative variables, and microenvironmental influences beyond what is captured in billing datasets. This study’s goal of developing a predictive machine learning model for capsular contracture using only patient-specific history factors to assist practicing surgeons in prognostication is particularly challenging. Although our model is the largest of its kind for this purpose, few variables demonstrated sufficient effect size to meaningfully influence clinical risk stratification. Bavaro et al. (*N* = 59, 28 with capsular contracture) and Chen et al. (*N* = 209, 144 with capsular contracture) leveraged machine learning to find predictors of capsular contracture [17, 18]. Bavaro and colleagues’ analysis investigated a postmastectomy cohort that only included breast cancer patients who underwent radiotherapy treatment but did include variables that were not available to us such as estrogen and progesterone receptor status, ki67 proliferation status, and histological characteristics. The random forest model in their study determined the following three features with the highest frequency on a ten-fold cross-validation scheme: (1) grading, (2) progesterone receptor status, and (3) lymph-node status (N0-N3). Chen and colleagues utilized seven machine learning models and found the neural network model achieved the best performance metrics. Their results from this study were consistent with the previous literature that prior irradiation and mesh type were two of the strongest predictors for capsular contracture [18]. Their analysis also found that higher age and dual plane implant placement increase risk, whereas a larger nipple-inframammary fold distance was slightly protective in development of capsular contracture (i.e., smaller native breast dimensions appear to increase the incidence of capsular contracture). This led them to suggest that a tighter pocket can lead to greater mechanical stresses, forces that have been evaluated by Fraldi et al. in finite element analysis [31]. Montemurro et al. (*N* = 1462, 44 with capsular contracture) used classification and regression tree (CART) analysis determined that higher BMI, preoperative bra-cup size larger than A, and higher age were the most predictive factors for development of capsular contracture [32]. Naoum and colleagues (*N* = 1617) developed a machine learning nomogram for individualized risk for postoperative complications after mastectomy, including capsular contracture requiring capsulotomy with a number of predictors ranging from demographics to tumor biology and treatment information. Studies such as Chen et al.’s and Naoum et al.’s provide examples of clinically useful risk stratification based on model-generated assessments of different clinical features associated with capsular contracture. Moreover, their investigations reinforce the importance of access to granular and institutional-level plastic and reconstructive surgery variables (e.g., mesh use, incision type, implant placement, lymph-node status, etc.).

In an effort to quantify the impact of a prior diagnosis of capsular contracture in our model, we removed it from the model and the accuracy decreased by 0.0011%. When removing prior irradiation as a feature, the model accuracy decreased by 0.00014%. These findings illustrate that statistical significance does not necessarily translate into meaningful predictive performance.

A critical distinction highlighted by our findings is the difference between statistical association and predictive utility. While prior capsular contracture and irradiation demonstrated significant associations with capsular contracture development, their inclusion produced only negligible improvements in model performance. Consequently, while contributory factors may be identifiable in individual patients, capsular contracture risk may not be adequately captured by historical administrative data alone and likely reflects complex biological and procedural interactions not measurable within claims datasets. Failure of prediction in this context should not be interpreted as absence of causation, but rather as evidence that meaningful risk likely resides in variables not captured within administrative data.

While there have been a myriad of surgical techniques and alternatives developed in an effort to reduce capsular contracture, autologous reconstruction eliminates implant-related capsular contracture risk, though it introduces its own distinct complication profile [33]. Machine learning models for capsular contracture looking from the basic science lens using RNA sequencing and tissue samples may reveal more about the potential underlying mechanism and progression [34]. Cellular-level insights have tended toward understanding the mechanisms behind capsular contracture [35]. Future studies can tune models with patient populations and data that combine the strengths of our study and the aforementioned machine learning analyses for capsular contracture prediction. Investigating the influence of antibiotic irrigation, acellular dermal matrix placement, and leukotriene inhibitor administration on capsular contracture development in this cohort will provide additional knowledge on the complex drivers of contracture formation. With these additional crucial variables, the analytical power of machine learning will most likely yield a more clinically relevant predictive model.

### Limitations

This study leverages machine learning to determine risk factors for capsular contracture with the largest sample size of capsular contracture patients in existing literature. However, this study is limited by its reliance on a national claims database and its retrospective nature. Given the dataset’s exclusion of many important patient and provider-specific details, we are unable to investigate a number of risk factors that have been linked to capsular contracture. Additionally, the external validity of our findings may be limited as we relied on administrative claims data that are largely derived from a private insurer. Patients who did not experience capsular contracture or other implant-related complications may have been less likely to return for follow-up care, leading to selection bias. Studies that use more strict definitions of capsular contracture such as requiring capsulotomy or surgical intervention may yield different findings and not account for certain patients who refused another operation [36]. Moreover, we are unable to investigate surgeon-specific contributors and surgical patterns that could contribute to capsular contracture. These findings underscore a broader limitation of claims-based machine learning in surgical outcome prediction. Large datasets do not inherently confer high predictive resolution when critical biologic and procedural variables remain unmeasured. Future research should investigate these other factors on a national scale, which can provide a larger and more generalizable patient population. Additionally, analysis of subgroups such as patients with increased irradiation may reveal unique clusters at risk of capsular contracture. Investigating the impact on quality of life and challenges faced with capsular contracture should likewise be further explored through mixed-methods analyses.

## Conclusions

Our model found that medical history alone, as captured by ICD and CPT codes in administrative claims data, demonstrated limited predictive utility for capsular contracture development. While previous associations between capsular contracture and aspects of the patient history were upheld, their clinical predictive utility appears limited.
